# Meaning and challenges in the practice of multiple therapeutic massage modalities: a combined methods study

**DOI:** 10.1186/1472-6882-11-75

**Published:** 2011-09-20

**Authors:** Antony J Porcino, Heather S Boon, Stacey A Page, Marja J Verhoef

**Affiliations:** 1 Department of Community Health Sciences, Faculty of Medicine, University of Calgary, Calgary, Canada; 2 Leslie Dan Faculty of Pharmacy, University of Toronto, Toronto, Canada; 3 Office of Medical Bioethics, Faculty of Medicine, University of Calgary, Calgary, Canada

## Abstract

**Background:**

Therapeutic massage and bodywork (TMB) practitioners are predominantly trained in programs that are not uniformly standardized, and in variable combinations of therapies. To date no studies have explored this variability in training and how this affects clinical practice.

**Methods:**

Combined methods, consisting of a quantitative, population-based survey and qualitative interviews with practitioners trained in multiple therapies, were used to explore the training and practice of TMB practitioners in Alberta, Canada.

**Results:**

Of the 5242 distributed surveys, 791 were returned (15.1%). Practitioners were predominantly female (91.7%), worked in a range of environments, primarily private (44.4%) and home clinics (35.4%), and were not significantly different from other surveyed massage therapist populations. Seventy-seven distinct TMB therapies were identified. Most practitioners were trained in two or more therapies (94.4%), with a median of 8 and range of 40 therapies. Training programs varied widely in number and type of TMB components, training length, or both. Nineteen interviews were conducted. Participants described highly variable training backgrounds, resulting in practitioners learning unique combinations of therapy techniques. All practitioners reported providing individualized patient treatment based on a responsive feedback process throughout practice that they described as being critical to appropriately address the needs of patients. They also felt that research treatment protocols were different from clinical practice because researchers do not usually sufficiently acknowledge the individualized nature of TMB care provision.

**Conclusions:**

The training received, the number of therapies trained in, and the practice descriptors of TMB practitioners are all highly variable. In addition, clinical experience and continuing education may further alter or enhance treatment techniques. Practitioners individualize each patient's treatment through a highly adaptive process. Therefore, treatment provision is likely unique to each practitioner. These results may be of interest to researchers considering similar practice issues in other professions. The use of a combined-methods design effectively captured this complexity of TMB practice. TMB research needs to consider research approaches that can capture or adapt to the individualized nature of practice.

## Background

Therapeutic massage bodywork (TMB) describes any treatment therapy that uses one or more massage techniques (kneading, stroking, pressing, vibrating, holding, etc.) of the soft tissues, viscera, and joints to achieve therapeutic effects. About 170 TMB therapies and variants (e.g., 3 different-differently named variants of Shiatsu) have been recognized in North America, with most of those available in Canada [1]. Of those, 25 are proprietary and trademarked, such as Trager™ and Onsen™, with tightly controlled training standards. The remaining TMB therapies, including reflexology, acupressure, and massage therapy, have not been uniformly standardized with respect to their definitions, training components or competencies (which can vary in training length and content by jurisdiction or school decisions), or regulation [2]. Longer and more advanced training programs may include a diverse mixture of introductory and full competency TMB and non-TMB therapies. Of the TMB therapies, massage therapy (describing basic Swedish to advanced "therapeutic" or "remedial" massage therapy) is the most commonly available and researched form in North America. While massage therapy is regulated in three Canadian provinces (but not the province of this study) and many U.S. states, other TMB therapies are not.

The term "training program" in this article may refer to any of the following *types *of education: apprenticeship; introductions to courses; training courses (certificate programs of a few hours to hundreds of hours) focused on a single therapy; extensive education programs (certificate or diploma programs that run from about 50 hours to 3000 hours) that may include one or more types of therapies and which may also include introductions to additional therapies; or self-study. Many TMB therapies can be learned through more than one of those routes. Within these training programs, the practitioners learn the therapies' *techniques*, the building blocks of therapy application.

Published TMB studies vary widely in terms of the specifics of the intervention(s) provided as well as the type of outcomes assessed. Often, the results of specific intervention studies do not extend beyond identifying general effects such as stress reduction or change in mood state, or are inconclusive. Few published TMB articles discuss whether the lack of conclusive results arises from using inadequate research methods or outcomes, conjecture, or presupposition caused by (1) lack of comprehension of the myriad forms of TMB; (2) assumptions regarding the definition of a given TMB therapy; or (3) not accommodating the normal adaptation of protocols (assessment and treatment) that are used in clinical TMB practice. An unpublished review of published TMB research by the principal investigator indicates that few studies: (1) report practitioner credentials, which may vary enormously; and (2) discuss the potential impact of practitioner variability on the results. Multiple-therapy training may potentially blur the identity of specific treatments, which causes practice under the name of a specific therapy to be blurred as well. Multiple-therapy training also may increase practitioner variability in treatment provision. Increasing understanding of the training and practice of TMB will facilitate the undertaking and interpretation of research in TMB. Therefore the purpose of this study was to: (1) document the scope of training and practice of manual therapy providers in Alberta, and (2) assess how training in, and provision of, multiple therapies may affect clinical practice. The broad focus of TMB in this study was used to capture information about how practitioners practice in real life.

## Methods

Clinical practice is complex involving many inter-related factors. A combined methods design, using both quantitative and qualitative methods of data collection and analysis in a single study, is ideally suited to capture this complexity [3]. A quantitative survey and semi-structured qualitative interviews were used to gather the data. The survey assessed the scope and length of practitioner training and basic practice characteristics (e.g. place, type of practice, focus of practice treatment and general population treated). Quantitative inquiry does not usually reveal *how *practitioners' practice characteristics or cumulative training affect treatment provision and decision-making, and thus cannot be used to *understand *actual treatment provision from the practitioners' perspectives. Therefore, qualitative interviews were used to supplement and enhance understanding of TMB practice. The interviews focused on: (1) how being trained in multiple therapies affects the practice and integration of treatments, and (2) whether practitioners who regularly combine techniques from multiple therapies can consciously isolate and dissociate specific techniques if asked to do so for a given research protocol.

The Conjoint Health Research Ethics Board at the University of Calgary granted ethics approval for this study. Personal identifiers have been removed or disguised to preserve anonymity.

### Data Collection

#### Questionnaires

Questionnaire development began with a pilot project to assess Alberta TMB practitioners' interest in participation in survey research of their professional practice [4]. Based on the results we developed a four-page questionnaire, informed by previously used massage therapist questionnaires [5-8]. The questionnaire sections comprise work environment descriptors, education and current practice, and practitioner demographics (copy available on request). The questionnaire went through two rounds of pretesting, with ten different TMB practitioners per round. The mailed questionnaire package included the questionnaire, a self-addressed stamped return envelope and a cover letter explaining the participation process, consent and privacy information, and a notice of a draw for a gift certificate for all practitioners returning their completed questionnaire. Consent to participate was implied if a completed survey was returned.

Alberta, Canada has a high number of TMB practitioners (> 5000), practicing a wide variety of TMB therapies, none of which are regulated. Twenty-two TMB organizations with members in Alberta were identified (list available on request). The four largest associations, the Natural Health Practitioners of Canada (NHPC), the Massage Therapist Association of Alberta (MTAA), the Alberta Registered Massage Therapists Society (ARMTS), and the Examining Board of Natural Medicine Practitioners (EBNMP) distributed the questionnaires on our behalf. Members of the remaining smaller associations were contacted through their on-line membership directories. In this process 5233 eligible practitioners were identified. Additionally, urban and rural spas were contacted to identify practitioners not affiliated with any organization. Managers at three spas distributed questionnaires to 16 TMB providers whom they believed had no organizational affiliation. We contacted the spa managers to verify questionnaire distribution. Whenever possible, an email pre-notification of the questionnaire was sent to questionnaire recipients as well as two follow-up emails, at two weeks and four weeks after the questionnaire mail out. Of the 5249 surveys distributed, seven were returned as undeliverable, (final n = 5242).

Statistical analysis was done using PSAW Statistics 17.0.2 [9] or R (open-source computing language for statistics) [10].

#### Interviews

Practitioners providing more than one TMB therapy were invited to take part in an interview through completing and submitting the volunteer contact form provided in the questionnaire package. The form assessed participants' gender, municipality population (later categorized into urban, semi-urban, and rural), work environment (clinic type(s)), and the therapies they practiced. These categories were used to purposively select the interview participants and allow for maximum variation. The volunteering form mentioned recruiting twenty-five participants; two hundred eighty-three practitioners volunteered for interviews. As male practitioners and non-massage therapists are a small minority in the total TMB population, they were oversampled to explore differences in perspectives possibly influenced by these characteristics. Each interviewed volunteer received a $40 honorarium. Practitioners not interviewed were thanked for volunteering after interviewing was complete.

The interview guide (Table 1) was based on discussions of the principal investigator with TMB practitioners, as well as his personal experience as a multiple-therapy trained TMB practitioner. He conducted all interviews, after obtaining informed consent. The interviews were in-person or by phone and lasted between 30 and 70 minutes. They were audio recorded and transcribed verbatim. Field notes were made at the time of the interviews.

**Table 1:** Interview Guide (final version)

1.	Could you briefly describe the manual therapy trainings that you have taken? We'll get to the details of them later.
2.	I'd like to get a little more depth on each of those now. Can we start with the first training you did. (prompt for reasons for that training, what it included, how long, practicum/cases studies and clinic time. Importance in practice now.)

3.	What about the next trainings you took? (prompt for reasons on why chosen, etc. Importance in practice now.)

4.	Did practice setting influence your choice of trainings?

5.	Did the initial training influence your style or current approach to your work?

6.	How do you use these therapies in your practice? (prompt for defining separation or mixing of therapies, any specific training on combining, attitudes, concerns, reasons, etc.)

7.	How do you choose which therapies to use together? What are the influences on your decision to use one technique or therapy over another?

8.	What forms of feedback do you use? How do you know when you are done in a specific area or using a specific technique/therapy?

9.	What was your process for learning how to use therapies together like this?

10.	Have some techniques or your experience changed the way you practice other techniques? Is this common for you? In what ways?

11.	Do you think that your later training and experience has changed you such that you could no longer offer your modalities as purely as when you first learned them? Could you provide a pure therapy if you had to?

12.	If you are combining therapies like this, how do you negotiate consent?

13.	Given what we've been discussing, what do you think about the idea of using a set routine for therapy × in a research project. Does it matter that switching/blending therapies might make it hard to research or evaluate what you do? (If time, explore a bit more about the use of evidence or perceived barriers for use in their practice.)

14.	Do you think that research and regulation are linked?

15.	My final question is from a result in the questionnaire part of the project where I asked if you treat people who cannot perform activities of daily living without your treatments. I'd like to get a sense of your understanding of what "activities of daily living" means.

16.	Is there anything else about the decisions, use, or training in therapies that you'd like me to know before we wrap up?

The computer program ATLAS.ti [11], was used to organize and assist content analysis of the qualitative data. Content analysis involves a straight reading of the data, comparing, organizing, and linking concepts and ideas (labelled with representative codes) within and across the interviews [12,13]. As analysis progresses, the coding scheme is progressively modified and refined. In our study, data analysis was ongoing throughout data collection. The interview guide was modified based on the first two interviews and further refined after the tenth, to better explore the developing material. Interviewing continued until data saturation was reached, the point at which new data did not contribute new ideas, concepts, or distinct variations to the findings [14].

## Results

### Questionnaires

#### Response rate and Demographics

Seven hundred ninety-one completed questionnaires were returned, a 15.1% return rate, with 57% respondents from the NHPC, 14% from the MTAA, 6% from the ARMTS, and 24% who did not indicate their affiliation. Comments on returned questionnaires indicate that the response rate was impacted by the summer distribution and concerns that the questionnaire would be used for the purpose of regulating massage therapy in Alberta. Table 2 compares this survey's results to previously published demographic surveys of the Natural Health Practitioners of Canada (NHPC) (pan-Canada survey of the massage therapy members) [6], the College of Massage Therapists of Ontario (CMTO) (province of Ontario, Canada, Registered Massage Therapists survey) [5], and the American Massage Therapy Association (AMTA) (pan-U.S.A. survey of massage therapy members) [15]. Despite the lower response rate of the present survey, there were no significant differences between the demographics in the surveys' samples.

**Table 2:** Demographic characteristics, and comparison to past surveys

Question	Category	This Survey	NHPC [6]	**CMTO **[5]	AMTA [15]	*X*^2 ^(df), significance
Practitioner gender (%)	Male	8.3	14.1	17	15	3.562 (3), p < 0.313
		
	Female	91.7	85.9	83	85	

Years in practice (mean years)		8.3 (s.d. 6.2) (range: 0 to 37 yrs)	NP*	5.5	7	0.566 (2), p = 0.753

Mean Hours Worked with client (mean hours)		20.5 (sd: 11.6, range 2 to 80)	18.2	18.9	20	0.168 (3) p = 0.983

Top three work settings: Total/Primary** (%)	Private clinic	44.0/32.2	41.8/NP*	46/NP*	NC*	3.59(4), p = 0.464
		
	Home clinic	34.3/29.7	42.2/NP*	25/NP*	NC*	
		
	Outcalls	29.7/8.6	32.1/NP*	29/NP*	NC*	

Municipality size (%)	Rural/small town settings (under 50,000)	38.8	NC*	NP*	NP*	
		
	Small cities (50,000 to 100,000)	15.3	NC*	NP*	NP*	
	
	Cities over 100,000 population	45.8	49.6	NP*	NP*	z test of proportions: z = 1.383; p = 0.168

Return rate (%)		15.1	39.4***	18.2	NP*	14.437 (2); p < 0.001

* NP = information not published; NC = information was published, but the categories were not compatible. ** "Total" is based on all places of work per practitioner, "primary" is a reduction to their single place of the most work. ***included follow-up phone calls to increase participation.

#### TMB therapies identified

Respondents were trained in 62 out of the 65 therapies listed in the questionnaire (no practitioners of Aston Patterning, Looyen Work, or Mitzvah Technique). An additional 15 unique TMB therapies, and 36 non-TMB therapies (e.g., energy work, shamanism, counselling, herbology, movement and stretching therapies, acupuncture) were identified in the 'other' category. Of the total 77 TMB therapies, 22 (Table 3) have been taught to more than 10% of the respondents (complete list of TMB therapies practiced available on request).

**Table 3:** TMBs identified during the project practiced by 10% or more of respondents

massage therapy (Western)	89.40%	TMJ therapy (temporomandibular joint therapy)	35.7
Swedish/spa massage	63.2	hot/cold stones massage	30.1
trigger point therapy	58.4	Craniosacral™	27.3
maternal/pregnancy massage	52.7	or cranial sacral therapy	
sports massage	45.9	aromatherapy	22.1
chair massage	45.4	acupressure	21.9
myofascial release	44.5	geriatric massage	15.5
lymphatic drainage massage or manual lymph drainage	43.2	pædiatric massage	15.0
		shiatsu	12.3
hydrotherapy	43.1	Neuromuscular Technique	12.0
reflexology	38.2	Visceral Manipulation™	11.5
PNF (proprio-neuromuscular facilitation)	36.4	Thai Massage/Thai yoga/nuad bo-rarn	10.6

#### TMB Training

**Figure 1:**
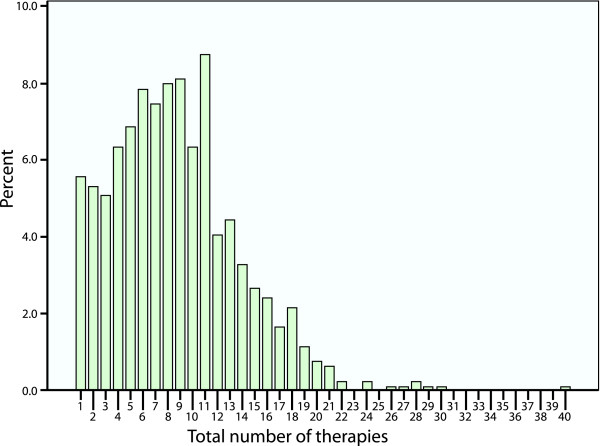
**Total number of therapies in which a practitioner has trained**.

Most practitioners (94.4%) are trained in more than one therapy, with a range of 1 to 40 therapies, and a median of 8 therapies (Figure 1). Of the 77 therapies identified, practitioners indicated that for 51 of those therapies, the training programs usually incorporated one or more (median of 3, range 1 to 17) additional therapies. The correlation (r = 0.115, p = 0.001) between number of years in practice and number of therapies trained in is low.

#### Training programs

Participants listed a total of 2,477 training programs with one or more TMB components. Length of the training programs varied widely, with no standard length for non-trademarked therapies. Their minimum training length ranged from 1 to 50 hours, with maximum hours ranging from 100 to 4,000. The shorter lengths for some therapies may have been introductory courses providing rudimentary training in some of the therapies' techniques; the questionnaire did not address the extent and depth of a training program. Most trademarked therapies had narrow ranges of training program length, like Hellerwork Structural Integration™ with a range of 1200-1250 hours.

On the questionnaire, respondents provided detailed therapy components for 856 training programs that included two or more TMB therapies. Massage therapy training programs were the most common (504 out of 856), with a median of four additional therapies in the training programs. For 641 of the 856 training programs, training program length was provided, which allowed checking for possible similar training programs between practitioners. Of those 641 training programs, 622 were unique programs.

Fifty-nine different TMB therapies were identified within the 856 multiple therapy training programs. Of the 12 therapies that appear in 10% or more of the training programs (Table 4), 10 are specialized techniques associated with the practice of massage therapy, either specific approaches (e.g., myofascial release, hydrotherapy) or for specific populations (e.g., sports massage, maternal massage).

**Table 4:** Additional TMB components included in more than 10% of TMB training programs

TMB Training Component	% of TMB Trainings Including the Component
Trigger point therapy*	38.6

Swedish/spa massage*	35.5

maternal/pregnancy massage	31.4

hydrotherapy*	28.5

chair massage	28.3

sports massage	26.3

manual lymph drainage	23.8

myofascial release	23.7

PNF	22.1

TMJ therapy	20.7

aromatherapy*	11.9

acupressure	11.3

*expected as part of a massage therapy training program, based on a review of massage therapy schools and common competency documents

### Interviews

The 19 interviewees indicated that they practiced between two and ten therapies on their volunteer form. During the interviews most practitioners described being trained in a greater number of therapies. Many participants also described taking introductory courses for additional therapies in which techniques from those therapies are sampled, as well as taking training in non-TMB therapies. Descriptors of the participants are included in Table 5. Number of years in practice was not a selection criterion for being interviewed, but it is included in Table 5 to show the range of experience covered by the participants.

**Table 5:** Interview participant characteristics, including reported therapies trained in

Gender	F = 15; M = 4
Work setting (n, not exclusive)	Shared clinic (4), private clinic (6), home clinic (4), salon (1), fitness club (1), spa (4), chiropractic clinic (2), medical clinic (1), outcalls (1)

Years in practice	3 to > 30 years

Number of TMB therapies trained in (including TMB course components)	Mean 8, range 1 - 16

Non-massage therapists	2; a third was trained in but did not practice massage therapy

Minimum number of introductory courses to therapies*	Mean 2, range 0 - 5

Number who also practice non-TMB therapies** (n)	12

• if therapists indicated generic introductory courses (e.g., "stuff at conferences"), but not the quantity, they were conservatively counted as one course. **includes devices, bio-energy treatments (e.g., Reiki), nutrition, ingested/topical products, systems approaches (shamanism, counselling).

Interview participants expressed complex and widely different responses to the interview questions. Four key themes emerged from the interviews: 1) the complexity of career and training paths; 2) all treatment is individualized; 3) the practice of therapies evolves over time; and 4) clinical practice and research treatment protocols are different. The first three have components that are relevant to describing the training and practice of TMB practitioners. The fourth theme describes why practitioners reference their clinical experience to distinguish between clinical practice and research-protocol treatments. Interview results from the purposefully oversampled male and non-massage therapist populations compared to the interview results of females and massage therapists, respectively, did not reveal any differences.

#### Theme 1: Career and training paths are complex

A number of career and training factors emerged in the interviews related to: the practitioner's vision of their work before they began their training; the type of practice environment they desired; the availability, time, and cost of training programs; and the pressures that affected subsequent training choices. Participants followed training pathways that were quite variable right from the start of their careers.

Entry into a TMB profession sometimes came from a long-time desire, or the realization that they were finally coming "home" to the profession, often after receiving some TMB or taking an introductory course. For others, it was a progression from previous employment, or an opportunity that enabled a switch into a new profession.

"My nurse friend said..., 'You really are in the wrong profession. ... you should do it [massage]' and got me an interview with the school. And when I did my first body I knew I had come home." (Practitioner 9)

Some practitioners had pre-conceived ideas of what the style of their first or primary training should be, e.g., focused on injury treatment and prevention relative to general health and well-being treatments, focused on one or a few specific, related TMB therapies, or wanting a program that was "holistic," incorporating multiple therapies and perspectives. Others instead chose their training programs for pragmatic reasons such as availability or because they could accommodate the training program schedule.

"I found this program in Medicine Hat that you could get the reflexology along with the massage and a whole whack of other stuff, and decided I would give it a try." (Practitioner 3)

Many training programs incorporate two or more therapies. Several practitioners talked about the inclusion of some "extra" introductory versions of therapies added to their primary therapy training program(s), giving them a couple of extra techniques, or a "taster" of the other therapies that they could then pursue at a later date. They often incorporate these introductory courses' techniques into their daily practices, but do not practice under the name of those therapies.

All the interviewed practitioners had taken more training after completing their initial training program. For all of them, the trend was to train in an increasingly diverse and often complex set of therapies over time. They spoke of these training choices as pursuing ideas and therapies of personal interest. This could be to refine or expand skills within their current treatment framework (e.g., remedial service), or to branch out to incorporate completely new therapy forms.

"I often took classes because I felt I needed more, 'cause I didn't have everything. When I first took massage therapy, I was ready to heal the world... And it doesn't. I mean, it's a really nice thing to do, but massage works on muscle, and muscle isn't the only cause of people's pain and dysfunction in this world." (Practitioner 10)

These additional therapies are often referred to as added "tools in the toolbox." The importance of the toolbox concept became clear as practitioners talked about how and why each treatment they provide is individualized (see also Theme 2 below).

"... and then I just go through my tool kit and say okay this is what would work best for that. That's how I fit things together." (Practitioner 4)

#### Theme 2: All treatment is individualized

The drawing on tools--the many therapies and techniques practitioners have learned--is an important process of individualizing a patient's treatment. Practitioners described three increasing levels of specificity in the individualization of treatment delivery: 1) the *initial treatment plan*; 2) *treatment plan variation*; and 3) *within-therapy variation*.

At the first level, an initial *treatment plan *is developed based on the treatment goals, which come from initial assessments (visual, testing, palpation) as well as dialogue with the clients about their goals, needs, and experiences. A treatment plan outlines the therapeutic intent(s) and treatment(s) for the current session and will map out the planned treatment progression for subsequent sessions, though a reassessment will occur at the start of each subsequent session.

"I start picking up the cues about how they [the patients] are functioning right from the beginning... whatever levels they're describing at: 'My shoulder is painful.' 'It happens when I'm doing these particular things.' ... I watch how their body is in space and I palpate to see what that feels like as they move those parts of the body that we're paying attention to at any particular time and I have certain set of movement check-ins that I do with people... then the next level that I work with, I check in with touch to find out exactly what is going on [in the person's structure]..." (Practitioner 14)

The second level of individualization is *treatment plan variation*, which occurs throughout every treatment session. Complex feedback loops based on palpation (tissue texture, temperature, pliability or tone), visual cues (pain, motion or tension changes, breath patterns), verbal feedback from patients, intuition, and the pressure of time frame are used to gauge the progress of the treatment at any moment. These cues inform awareness of the treatment progress and choices at that moment, suggesting either to continue, to change therapy techniques, or move to a different therapy as they continue to work. They may also pause treatment to do a more deliberate reassessment before continuing treatment. All interviewees, regardless of whether they kept to only one therapy during a treatment (two interviewees) or integrated several therapies into the treatment plan (17 interviewees), described modifying their treatment plans based on in-the-moment assessment.

"If I've been working there for a while and I'm not getting any releases there, then I go from the microscopic, you know, looking at that hip for example, and I broaden my scope and go to macroscopic, and I start looking at what's going on in the low back, what's going on in the pelvis area--on the front of the pelvis--that could be affecting what's going on in the hip. Or I might need to go down into the leg. So just broadening my scope, and usually the body will draw me to the next place that needs to be addressed." (Practitioner 12)

"Sometimes I've kicked in three different things back-to-back. Depends on how the body is releasing." (Practitioner 10)

The final layer of individualizing is *within-therapy variation*. Occurring at any moment during a session, this may be a spontaneous or planned shift in a particular therapy's technique, or the integration of another therapy's technique within the therapy the practitioner is currently applying so as to better address the perceived treatment need. This level includes the described variations on "listening to the hands," where practitioners let their hands spontaneously react to tissue cues.

"The more I learn the more I know I don't know. (laughs) My hands really have to ... [interrupting herself] I listen to my hands. My hands tell me where to go next, and they don't care what definition the technique is listed under." (Practitioner 10)

Practitioners consider the strength and healing possibilities in their work to be at the second and third levels of individualizing treatment.

"Palpation is probably the most paramount ingredient to use during the course of the treatment. You're evaluating throughout the course of treatment. You're evaluating the tissue, the texture of the tone, everything like that in the muscle, determining how it's responding." (Practitioner 5)

Some had critical words for practitioners who would tend to practice using routine patterns with little adaptation or individualizing.

"I mean, you know this is the most important thing actually. I mean if you just follow a stupid protocol, you know we just call these people the skin pushers." (Practitioner 11)

The importance of this complex, adaptive treatment process based on continual feedback from multiple information sources was echoed in ideas expressed about TMB research based on restrictive protocols compared to clinical practice (Theme 4).

#### Theme 3: Therapy provision will evolve over time

Discussions of within-therapy variation of technique led to a critical question of exploration: does a given therapy, as practiced, change over time from the accumulating experience of a practitioner, including influences from the multiple-therapy integration that happens as part of the process of individualizing patient care? The practitioners expressed two primary, contrary opinions about this. Most asserted that it would be easy to provide a therapy uninfluenced by techniques from other therapies they had learned, or at least with disciplined focus they could do so.

"I think definitely who I am today, all of that has influenced me. But I also know that if somebody said to me, 'I want a straight fascial work' or 'I want a straight sport massage work' or 'I want a straight Swedish massage work', I could do that. I could pull them apart and still do them." (Practitioner 1)

However, they all acknowledged that practice becomes refined due to practice experience, exposure to different therapy techniques over time, or both, making every therapist's application unique. As Practitioner 11 put it, referring to the idea of a generic practitioner practicing a pure, as-trained therapy, "they could, but you know they haven't learned then." Several highly self-reflective practitioners speculated that no one fundamentally practices an unaltered therapy. They postulated that any TMB application is likely permanently altered due to practice experience and alteration of perception or techniques from multiple TMB training programs, even if that alteration is not conscious.

"...my hands just can't operate at the gross [basic] level they used to for massage. When I'm doing a massage... sometimes I'm feeling the lymph and sometimes I'm feeling the energy... some type of an energy cyst, from the Craniosacral perspective. Or I'm feeling that the fluids are not moving from the lymphatic drainage [perspective]." (Practitioner 10)

#### Theme 4: Clinical practice and research treatment protocols are different

The individualization process underlies the fourth theme, *clinical practice treatments are different from the treatment protocols used in research*. Based on deduction from published research, practitioners insist there is a distinction between the two, which they dichotomize as either individualized clinical practice or pre-defined, restrictive research treatment protocols.

"Well, I think research is research and practice is practice. Research, you're setting out to find a specific thing. You're not trying to ...well, you *are *trying to help someone, but you're more about how this particular thing affects that person or that pathology or that injury. So you have to be consistent... you can't change it, or how do you know that it wasn't one of the other things, right?

Practice is a whole different thing. You're not there to prove to the client that this technique works. It either does or it doesn't, and if it doesn't you need to move onto something else, 'cause it's different for every person. So you're treating the person, whereas with research you're researching." (Practitioner 15)

Underlying these comments is a shared practitioner wariness of the clinical usefulness of research results. As described above, clinical practice treatment normally would be individualized to maximize therapeutic outcome. Commonly, applying a research protocol or using a single approach to a symptom is highly constrained; practitioners may not consider such a treatment process as appropriately responsive to what was occurring in the body. Therefore the relevance of treatments in research seems removed from everyday clinical practice.

"I think that when I've seen the early research that's been done with short stroke and all that kind of stuff for tension and pain management, I think that they are flawed because they do not take in [to account] tissue response. ...You would have to do proper assessment of the appropriateness of your approach for the person. As long as you provide massage or any other technique only as a set routine, you always miss the broader lived experience, the organism's response to what you're doing. There necessarily needs to be the capacity for ongoing assessment and adjustment of the treatment approach to the person's response to the treatment as part of getting a proper reading of whether it's doing what it should be doing." (Practitioner 14)

## Discussion

The results of this study present a complex view of the training and practice within the TMB professions, effectively revealed through the use of combined methods. Therapy training programs are highly variable in length and content, and most practitioners take additional education, resulting in few practicing with similar skill sets. The process of individualizing patient treatment explains how the myriad combinations of therapies are applied in clinical practice.

Given that manual therapies seem highly changeable, adaptable, and evolve differently with each practitioner, the question of what the application of a single therapy during a therapeutic session represents is a critical one that warrants further exploration with the TMB professions. Considering this study's combined quantitative and qualitative results reveals that almost all TMB practitioners 1) have training in multiple therapies, 2) use unique combinations of therapies and have unique experience, and 3) preferentially practice by individualizing treatment. This leads to the conclusion that most TMB treatments, even provided within the framework of a specific therapy, will be unique to the practitioner.

### The number of therapies trained in may be under-reported

There was a possible bias to under-reporting the number of therapies taught in multiple-therapy training programs in this survey. Some therapists reported that their two- to three-year education contained only a single therapy: massage therapy. However, the Canadian standard and published school curricula of these long, non-standardized programs indicate they provide training in multiple therapies. This could indicate that there is greater under-reporting than is recognized within the data. In addition, the number of therapies in which practitioners receive training will not represent all therapies used in practice. Some of the interviewed practitioners asked whether to discuss "introductions" to therapies within training programs or as part of continuing education opportunities, and some talked about self-education. As this training could affect practice, the potential impact was explored. The practitioners explained that while they may regularly use these additionally learned techniques during their practice, they do not consider themselves as having formally learned the therapy, and therefore did not report them in the survey question regarding the therapies in which they are trained. Hence the reported number of therapies the practitioners are trained in may actually under-represent the true total number of therapies or therapy techniques being used in practice.

### Skill sets vary widely between practitioners

The questionnaire results indicate high variability in program length and limited duplication in the multiple-therapy programs, implying that very few therapies have similar training programs. Few practitioners limit themselves to learning only one or two therapies. Additionally, most TMB practitioner associations require on-going education and upgrading of skills, which encourages learning a wide variety of therapies and techniques (e.g., NHPC, CMTO, Reflexology Association of Canada [16-18]). On their websites, many associations provide listings and internet links to a broad range of TMB training courses (e.g., NHPC, MTAA [19,20]). Over time, it seems likely that even practitioners of standardized therapies will acquire additional therapies and techniques and refine their skills through experience, therefore changing their techniques and their experience of applying therapies. Thus, while recent graduates of a program may acquire similar skills and techniques, through experience and later training, very divergent skill sets and idiosyncratic practice will evolve.

Of critical importance in the interviews was the disagreement between practitioners regarding the provision of pure "as trained" therapies. While some practitioners believed they could provide an "as trained" therapy, they also discussed how they had learned from experience, and most described having "refined" or "enhanced" their therapeutic skills via new awareness from other therapies' techniques or skills. This accords with the strong comments from other practitioners that the practice of therapies is likely irrevocably changed from practice experience and learning new therapies. It is unlikely that a researcher will find multiple practitioners who all practice any therapy in precisely the same way, or may be able to apply a protocol in precisely the same way. There is little mention of these issues in the TMB literature. The reporting of practitioner qualifications and expertise, along with intervention standardization and tailoring, are identified explicitly in the 2008 Consolidated Standards of Reporting Trials (CONSORT) Statement extension for Non-Pharmacological Treatment Interventions (internationally adopted publication guidelines for clinical trials) [21]. This inclusion indicates a growing awareness that practitioner variability may be affecting clinical trial results of many healthcare procedures, such as, "surgery, technical procedures (for example, angioplasty), implanted devices (for example, pacemakers), nonimplantable devices, rehabilitation, physiotherapy, behavioral therapy, psychotherapy, and complementary and alternative medicine" (page W60 [21]).

### The contrast of research and practice treatments

This lack of treatment process uniformity should be accommodated within a research project design or analysis for any therapy where variability in practitioner experience or cross-training is common. Practitioners made strong statements about the perceived differences between clinical practice treatments and those provided during the research process. They do not seem to value research results in practice, as it does not reflect how their therapies are *applied *in practice, implying that current research methods and knowledge translation are failing the TMB community. This phenomenon has been addressed by Schön [22], who reflects on the "artistry" of practice versus research in reflective-responsive professions, including similar health professions such as nursing and physiotherapy [22-24]. Given that traditional clinical trial research methods do not seem to effectively capture clinical practice, effectiveness and comparative research methods that may use practice-based adaptive protocols, and observational research, seem most likely to accommodate the realities of clinical practice as revealed in this study [25-27].

### Study limitations

Both the qualitative and quantitative data were internally consistent, and triangulated well. The primary limitation of this study is the survey's low response rate and therefore whether the survey's results are generalizable to TMB populations in general. A low response rate was somewhat expected given (1) the respondents' comments as described in the results section (summer distribution and concern regarding use of the survey results to influence massage therapy regulation), (2) the pilot project result that 23.7% of participants were not interested in or did not have the time to complete surveys longer than two pages, and (3) feedback from three North American massage therapy organization executives that "if you are getting a 15% response rate, you're doing well in this profession." Of importance, there is high concurrence between the demographics from different surveys (Table 2), suggesting similarity to other North American TMB populations.

A second concern is whether the practitioners responding to this survey differ from TMB practitioners in general, i.e., if non-respondents train in more, fewer, or different therapies, or have very different work habits or environments. The NHPC Membership, Credentialing, and Education Manager, Laura Finley (personal communication, June 1, 2011), confirmed that the survey results correspond to the NHPC Alberta TMB membership as well as its pan-Canada TMB membership regarding: (1) the vast majority of practitioners train in multiple programs and therapies, especially if the components of education programs and continuing education are considered, and (2) there is high variability in the training programs and in what therapies practitioners choose to learn. As well, the extreme variability within the survey practice and training program data suggests that a wide variety of practice variations have been captured in this survey. The interview data from the nineteen diverse practitioners were also highly congruent with the survey data. Therefore, even if a greater response rate had been achieved, the conclusions here would remain important considerations for research in the TMB professions.

## Conclusions

The training programs, number of therapies trained in, and practice descriptors of TMB practitioners are all highly variable. Further, with clinical experience and continuing education, therapy techniques will likely alter or will be enhanced, increasing the degree of individualized client care possible during practice. That on-going individualization process, at commencement and during treatment, is an essential element of a practitioner's practice.

### Implications for research

A concern arising from the data is that projects based on single therapy, non-adaptive protocols will likely continue to produce non-conclusive results for all but the most general of outcome effects such as reduction of stress or depression (two common positive TMB research outcomes) because of the high practitioner variability in training and experience, and the possibility that the strength of TMB treatments comes from their adaptive process. Therefore, TMB research design and results interpretation should include careful consideration of the limitations of implementing results from study designs that do not reflect the very complex reality of clinical practice. It also seems likely that issues of training, experience, and practice are not limited to the TMB professions. Complex systems methodology, based on mixed methods with their ability to capture the complex outcomes inherent in the practice of TMB, is recommended for TMB research. Comparative effectiveness research designs may best capture TMB treatment complexity, especially pragmatic trials and similar practice-based research methods that replicate daily practice within a controlled framework [25,26]. Preference trials, and observational research could also be used. These research designs have the potential to focus on real life practice and to capture the complexity of treatment packages.

## List of Abbreviations

AMTA: American Massage Therapy Association; ARMTS: Alberta Registered Massage Therapists Society; CMTO: College of Massage Therapists of Ontario; CONSORT: Consolidated Standards of Reporting Trials; EBNMP: Examining Board of Natural Medicine Practitioners; MTAA: Massage Therapist Association of Alberta; NHPC: Natural Health Practitioners of Canada; TMB: therapeutic massage bodywork.

## Competing interests

The authors declare that they have no competing interests.

## Authors' contributions

AP conceived of the study, participated in its design, carried out the data collection and primary analysis, and drafted the manuscript as part of his doctoral thesis. MJV participated in the design, oversaw the progress of the data collection, reviewed the data analysis, and helped draft the manuscript. HB and SP participated in designing the project, and editing manuscript drafts. All authors read and approved the final manuscript.

## Pre-publication history

The pre-publication history for this paper can be accessed here:

http://www.biomedcentral.com/1472-6882/11/75/prepub

## References

[B1] Stillerman E The encyclopedia of bodywork from acupressure to zone therapy 1996 1 New York, NY, U.S.A.: Facts On File

[B2] Porcino A Mah R MacDougall C Application for the regulation of Massage Therapy using a multi-category model in the province of Alberta 2006 Edmonton: Association of Massage Therapists and Wholistic Practitioners 1 385

[B3] Creswell JW Plano Clark VL Gutmann ML Hanson WE Tashakkori A, Teddlie C Advanced Mixed Methods Research Designs Handbook of Mixed Methods in Social and Behavioral Research 2003 Thousand Oaks, CA: Sage 209 240

[B4] Porcino A Verhoef M Alberta's CAM Manual Therapy Providers' Research Contact Preferences Coming of Age--Emerging Issues and New Directions in CAM Research: the Fourth Annual IN-CAM Symposium: 2007 2007 Vancouver, BC: Journal of Complementary and Integrative Medicine

[B5] Collis & Reed Research Report on the Massage Therapy Census 2003 - Membership Survey Report 2003 Toronto, ON: College of Massage Therapists of Ontario 67

[B6] Porcino A Highlights from the 2004 AMTWP Massage Therapist Survey Massage Therapy Canada 2005 Summer 2005 50 52

[B7] Sherman KJ Cherkin DC Kahn J Erro J Hrbek A Deyo RA Eisenberg DM A survey of training and practice patterns of massage therapists in two US states BMC Complementary and Alternative Medicine 2005 5 1 13 10.1186/1472-6882-5-13 15955245 PMC1182347

[B8] Montgomery LE Job Analysis of NCE-Level Therapeutic Massage and Bodywork Professionals, Conducted on behalf of NCBTMB 2003 Princeton, NJ: The Chauncey Group International 118

[B9] SPSS: an IBM Company PSAWStatistics (version 17.0.2) 2009 Chicago, IL: SPSS: an IBM Company computer program

[B10] R Development Core Team R: A Language and Environment for Statistical Computing (version 2.10.0) 2010 Vienna, Austria: R Foundation for Statistical Computing computer program

[B11] ATLAS.ti Scientific SoftwareDevelopment GmbH ATLAS.ti - The Knowledge Workbench LM (version 6.0.0.1) 2009 Berlin, Germany: ATLAS.ti Scientific Software Development GmbH computer program

[B12] Sandelowski M Whatever happened to qualitative description? Research in Nursing & Health 2000 23 4 334 340 10.1002/1098-240X(200008)23:4<334::AID-NUR9>3.0.CO;2-G 10940958

[B13] Thorne S Interpretive Description 2008 1 Walnut Creek, CA: Left Coast Press

[B14] Strauss A Corbin J Basics of qualitative research: grounded theory procedures and techniques 1990 1 London, UK: Sage

[B15] American Massage Therapy Association 2010 Massage Therapy Industry Fact Sheet 2010 Evanston, IL: American Massage Therapy Association 4

[B16] NHPC Continued Competency Program Guide http://www.nhpcanada.org/centre-for-learning/continued-competency/

[B17] CMTO Guidelines for Continuing Education Units http://www.cmto.com/member/CEUNewGuide.htm

[B18] Continuing Education http://www.reflexolog.org/CEU.html

[B19] Learning Opportunities http://www.nhpcanada.org/centre-for-learning/learning-opportunities/

[B20] Continuing Education: Search upcoming courses http://www.mtaalberta.com/?page=120

[B21] Boutron I Moher D Altman D Schulz K Ravaud P The CONSORT Group Methods and Processes of the CONSORT Group: Example of an Extension for Trials Assessing Nonpharmacologic Treatments Annals of Internal Medicine 2008 148 4 W60 W66 18283201 10.7326/0003-4819-148-4-200802190-00008-w1

[B22] Schön DA The Reflective Practitioner 1984 1 New York, USA: Basic Books

[B23] Benner P From Novice to Expert: Excellence and Power in Clinical Nursing Practice 2000 Upper Saddle River, USA: Prentice Hall

[B24] Jensen GM Gwyer J Shepard KF Hack LM Expert practice in physical therapy Phys Ther 2000 80 1 28 10623958

[B25] MacPherson H Pragmatic clinical trials Complementary Therapies in Medicine 2004 12 2-3 136 140 10.1016/j.ctim.2004.07.043 15561524

[B26] Horn SD Gassaway J Practice-based evidence study design for comparative effectiveness research Med Care 2007 45 10 S50 17909384 10.1097/MLR.0b013e318070c07b

[B27] Patton MQ Qualitative Evaluation and Research Methods 2001 3 Thousand Oaks, USA: Sage Publications

